# Movement and Physical Activity in Early Childhood Education and Care Policies of Five Nordic Countries

**DOI:** 10.3390/ijerph182413226

**Published:** 2021-12-15

**Authors:** Ann-Christin Sollerhed, Line Grønholt Olesen, Karsten Froberg, Anne Soini, Arja Sääkslahti, Gudrún Kristjánsdóttir, Rúnar Vilhjálmsson, Ingunn Fjørtoft, Robert Larsen, Jan-Eric Ekberg

**Affiliations:** 1 Faculty of Teacher Education, Kristianstad University, 291 39 Kristianstad, Sweden; 2 Centre of Research in Childhood Health, Research Unit for Exercise Epidemiology, Department of Sports Science and Clinical Biomechanics, University of Southern Denmark, 5230 Odense, Denmark; lgolesen@health.sdu.dk (L.G.O.); kfroberg@health.sdu.dk (K.F.); 3 Department of Education, University of Jyväskylä, 40014 Jyväskylän, Finland; anne.j.soini@jyu.fi; 4 Faculty of Sport and Health Sciences, University of Jyväskylä, 40014 Jyväskylän, Finland; arja.saakslahti@jyu.fi; 5 Faculty of Nursing, University of Iceland, 101 Reykjavik, Iceland; gkrist@hi.is (G.K.); runarv@hi.is (R.V.); 6 Faculty of Humanities, Sports and Educational Science, University of South-Eastern Norway, 3672 Notodden, Norway; ingunn.fjortoft@usn.no (I.F.); robert.larsen@usn.no (R.L.); 7 Department of Sport Sciences, Malmö University, 211 19 Malmö, Sweden; jan-eric.ekberg@mau.se

**Keywords:** curriculum, education, movement, physical activity, preschool, early childhood education and care, children, Nordic

## Abstract

The purpose of this study was to examine the values of movement and physical activity (MoPA) using government policy documents (e.g., laws and curricula) on early childhood education and care (ECEC) from Denmark, Finland, Iceland, Norway, and Sweden. This descriptive, comparative study was designed based on curriculum theory and used word count and content analyses to identify similarities and differences in the occurrence of MoPA in the ECEC policies of Nordic countries. Seven terms were identified as MoPA-related in Nordic policy documents. These terms occurred in various content contexts: development, environment, expression, health and well-being, learning and play, albeit sparsely. MoPA was referred to as both a goal in and of itself and as a means of achieving other goals (e.g., learning or development in another area). Formulations specifically dedicated to MoPA as a goal were present in the Danish and Finnish curricula and, to some extent, also in the Norwegian curriculum, while the Icelandic and Swedish curricula mentioned MoPA mostly as a means. Findings indicated that MoPA, which is important for children’s development, health, and well-being, is a low-priority value, to varying degrees, in the ECEC policies enacted by Nordic countries and the guidance provided to educators and stakeholders therein is inexplicit.

## 1. Introduction

Children develop rapidly during early childhood, especially in the domain of movement and physical activity (MoPA). Motor development refers to physical growth and a child’s increasing ability to independently direct their MoPA and explore their environment [1], which is important for the development of other essential non-motor competencies including cognitive, communication and language, social and emotional skills [2]. MoPA impacts children’s overall development and their ability to interact through play [3]. Early childhood development lays the foundation for a lifetime of mental and physical health as well as for future academic achievement and overall well-being [4]. Children who do not participate regularly in motor skill-enriched activities may never reach their genetic potential for motor skill control, which underlies sustainable physical fitness later in life [5]. Early childhood development is currently receiving increasing attention and is included in the Sustainable Development Goals by World Health Organization (WHO), United Nations Children’s Fund (UNICEF) and Lancet Commission [6,7].

Fundamental movement skills (FMS) include stability, locomotor and object control skills, and are vital for motor development in children [8]. The benefits of PA and disadvantages of physical inactivity are well recognized [9] and global guidelines on PA for all age groups are published by the World Health Organization (WHO) [10]. Childhood PA and inactivity seem to track into adulthood [11,12,13]. The relationship between FMS in childhood and lifelong PA [14] highlights the importance of learning FMS in early childhood [15]. As most children attend early childhood education and care (ECEC), it is a well suited arena for developing FMS; this process depends on knowledge and motivation among teachers [16], which are often dictated by governmental and institutional curricula [17].

During the last century, the Nordic countries Denmark, Finland, Iceland, Norway, and Sweden established a welfare model, which includes developing an equal society and access to ECEC for all children. This means that children are the responsibility of not only the family but also of society, just like school education [18]. ECEC includes children up to school age in the Nordic countries [19]. Broström et al., (2018) conclude that almost all Nordic children between 1–5 years old spend a considerable portion of their daily lives in ECEC [18]. The Organization for Economic Co-operation and Development (OECD)’s ECEC policies address the needs of children between 0–8 years of age [20] as important and recognize the concept of ‘lifelong learning’. This strengthens ECEC as an educational institution, which now is considered to be the first stage of lifelong learning, important for laying the foundations for future learning [21]. Karila (2012) argues that ECEC is widely seen as an investment in the future [22]. The goal of lifelong learning is explicitly stated in most Nordic countries’ ECEC legislation [22,23,24] and has been implemented in the context of the welfare state and connected to welfare policy areas [22]. The Nordic countries are close geographically, but also culturally [25] and share many common beliefs and values. ECEC in the Nordic countries, sometimes called the “Nordic model”, has been a topic of global interest and used as a good example to follow by many countries [19].

Since a growing number of children attend ECEC, expectations that it will support the development and learning of the youngest children are high. ECEC are governed by different policies embodied in both laws and curricula. These formal binding governing documents are essentially a representation of the values of a society that serve to guide the work and organize the knowledge concerning ECEC. Engel et al. (2015) argue that the framework of a curriculum plays a key role in ensuring the quality of ECEC services [26]. The law and the curriculum reflect the knowledge that has been legitimized by society [27,28] and what should be educated [29]. Moreover, policy documents govern and guide different actors (e.g., teachers and politicians) [30] in organizing ECEC to optimize children’s development, and if matters are not presented conspicuously, it might be ignored and consequently affect the children’s daily life both in the short and long-term.

In this study, “curriculum theory” was used as a theoretical framework since the study sheds light on policy documents such as laws and curricula and how MoPA is valued in these documents and their limitations. According to Young (2013), curriculum theory concerns identifying curriculum constraints and what qualifies as knowledge, what knowledge is legitimized through curriculum formulations and the selection of knowledge in curriculum construction [27,31,32,33]. In curriculum theory, the curriculum is a concept that not only includes policy documents prescribed by the government but also includes the entire school system in which teaching, and learning occur. Deng and Luke (2018, p. 82) state that the “selection and organization of subject matter is one of the most basic, ubiquitous, and central moments of curriculum formation” [34]. These processes are influenced by different interests and desires, and curriculum settlements are often the result of many political and ideological compromises (ibid). According to Bernstein (2000), education is a field where struggles occur between different opinions and actors, competing to define the field and ultimately to determine what knowledge is to be counted as legitimate [27,35]. Young (2013, p. 110) argues that a “national curriculum should limit itself to the key concepts of the core subjects” and that the curriculum should guarantee that all children have the same and equal possibilities to develop an equivalent knowledge base regardless of background, conditions and location [31]. Curricula reflect the values and atmosphere of a society regarding how different tasks are approached, e.g., the role of ECEC. It guides the values, goals and contents of the work of early childhood educators and serves as a point of reference for teachers and schools [30]. In Nordic countries, the laws and curricula are binding documents and guides equal ECEC. These must uphold an obligation to provide equable (equal in quality) education and care (Educare). Linné (2015) argues that researching from a curriculum theory perspective means to be interested in the knowledge referred to, the content realized, and how transfer and valuation are implemented [36], and thus what knowledge is selected and valued. MoPA expressed for its own sake and value was considered to be a goal, while when expressed for the purpose of achieving other goals such as development in other areas [37,38], MoPA represented a means.

Comparative studies can shed light on what is unique to a countries’ culture and what is shared between cultures, which could be valuable for developing effective MoPA policies and education practices that support lifelong participation in MoPA in Nordic as well as other countries. Such studies have been published on Nordic ECEC policy documents; however, there are concerns about the quality and content of those conducted in Nordic countries [39,40], as well as their values of democracy, caring and competence [41], quality aspects of different curricula [2], the current and future directions of Nordic ECEC policy-making [22] and central dimensions and dilemmas in Nordic ECEC [18]. Other studies have been conducted between some Nordic countries and other countries, such as play and learning in Norway, Finland, China and Hong Kong [42] as well as the use of legislative documents to examine the sustainability of ECEC policies across different countries [43,44,45,46]. An international study investigated the national recommendations regarding PA for children under the age of 5 in 10 countries [47]. To the best of our knowledge, no comparative studies have been published on how MoPA is presented and valued in Nordic legislation and curricula.

Since MoPA has increasingly been flagged as an important issue within ECEC [48,49] and for sustainability [6,7] and health by the WHO [10], it is vital to investigate how MoPA is conceptualized in current ECEC policies in Nordic countries. Given that national laws and curricula are formal, state-governed, binding documents intended to guide and regulate ECEC that represent the values and priorities of a society and culture, these were the primary source material included in this study. Through a comparative study of how MoPA is represented and valued in these documents, the present study can shed light on and expand our understanding of how MoPA is valued in the Nordic countries.

The purpose of the present study was to identify and discuss the similarities and differences among the ECEC law and curricula adopted by the Nordic countries. Toward this end, the following research questions were formulated:

- Which terms related to movement and physical activity (MoPA) are present?
- In what content context do movement and physical activity (MoPA) related terms occur?
- Is movement and physical activity (MoPA) expressed for its own sake as a goal or as a means to achieving other goals?

### Organization of ECEC in Nordic Countries and Documents Included in the Study

Children in the Nordic countries start ECEC between 8 months and 1 year of age and start compulsory school between 5 and 6 years old. Different terms are used for ECEC in the five Nordic countries that need to be acknowledged when reading the laws and curricula included in the study (see Appendix A).

In Denmark, ECEC (dagtilbud) covers all daycare facilities including family daycare (0–2-year-olds), day nursery (0–2-year-olds) and kindergarten (2/3–5/6-year-olds). The year and month of typical kindergarten starting times vary within the Danish Municipalities. Compulsory school typically starts in August of the year the child turns 6 years old. Most daycare facilities are public. The percentage of children attending kindergartens (børnehave) is 97% whereas the percentage of children attending any daycare facility is 66% for those aged 0–2 years due to maternity leave (2017). ECEC is regulated by the Day-Care Facilities Act (Dagtilbudsloven nr. 2/2020, Chapter 2), and the Executive Order on pedagogical objectives and content in six curriculum themes (Bekendtgørelse om pædagogiske mål og indhold i seks læreplanstemaer 2018), one theme being `body, sense and movement’.

In Finland, ECEC encompasses children aged 0 to 7 years old. There are different ECEC centers, including municipal (73%) or private (28%), or family daycare (7%) (2018). Approximately 74% of the 1 to 6-year-old children participate in ECEC. The participation rate increases with age: under 1 (1%), 2 (66%) and 5 (89%)-year-old children. Upon turning six years old, all children start obligatory preschool (pre-primary school, esikoulu), and the school begins in the year when children turn seven. ECEC is regulated by law, by the Act on ECEC (Varhaiskasvatuslaki 540/2018), and the Curriculum for ECEC (Varhaiskasvatussuunnitelman perusteet 2018) determined by the government and Finnish National Agency for Education.

In Iceland, preschool (leikskóli) constitutes the first level of the education system and is attended by children below the compulsory school age, which usually starts when the child turns 6. Voluntarily and at the request of parents, preschools provide upbringing, care and education for children of preschool age, 1 to 5/6 years old. ECEC is regulated by the Preschool Act (Lög um leikskóla 90/2008). The Icelandic national curriculum–Guide for preschools, (Aðalnámskrá leikskóla–Almennur hluti 2011) contains the framework and conditions for learning and teaching based on the principles of existing laws, regulations and international conventions and is directed by the Ministry of Education. In 2019, 90% of children attended pre-schools, 75% of children in the age of 1 to 2 years and about 99% of children 3 to 5 years old.

In Norway, kindergarten (Barnehage) is voluntarily offered to all children 0/1–5/6 years of age. 93% (2020) of children aged 1 to 5 years attend kindergarten (March 2020: 85% age 1–2 years, 97% age 3–5 years). About 97% attend kindergarten full time. Kindergartens are divided into public and private, with about half of each type of kindergarten. All institutions are subject to common legislation and policy. ECEC is regulated by The Kindergarten Act (Barnehageloven 2021), a separate law for kindergartens administered by the Ministry of Education and Research. The law states the kindergartens’ responsibility for children’s welfare and rights, kindergarten practices, organization, and administration. The Framework Plan for Kindergartens (Rammeplan for barnehagen 2017) regulate content and tasks and is divided into nine chapters concerning core values, administration, organization, educational activities, disciplines, and practices. Compulsory school generally starts the year in which children turn 6.

In Sweden, preschool (förskola) refers to a voluntary form of schooling that occurs prior to compulsory school, which usually starts when the child turns 6. There are municipal (72%) and private (28%) preschools (2019), both regulated by the same national policy documents. The child attends preschool from the age of 1 until 5/6 years old. Almost 85% of children aged 1 to 5 attend preschools, and about 95% of children in the 4 to 5 age group attended preschools in 2020. ECEC is regulated by the Education Act (Skollag 2010), a law divided into 29 chapters concerning the entire school system. Chapter 8 covers preschool and, as such, was included in the present study. The Curriculum for the Preschool (Läroplan för förskolan 2018) is a regulatory national curriculum that formulates fundamental values, learning directives, goals and content together with specific responsibilities for staff and the head of the preschool.

## 2. Materials and Methods

**Figure 1:**
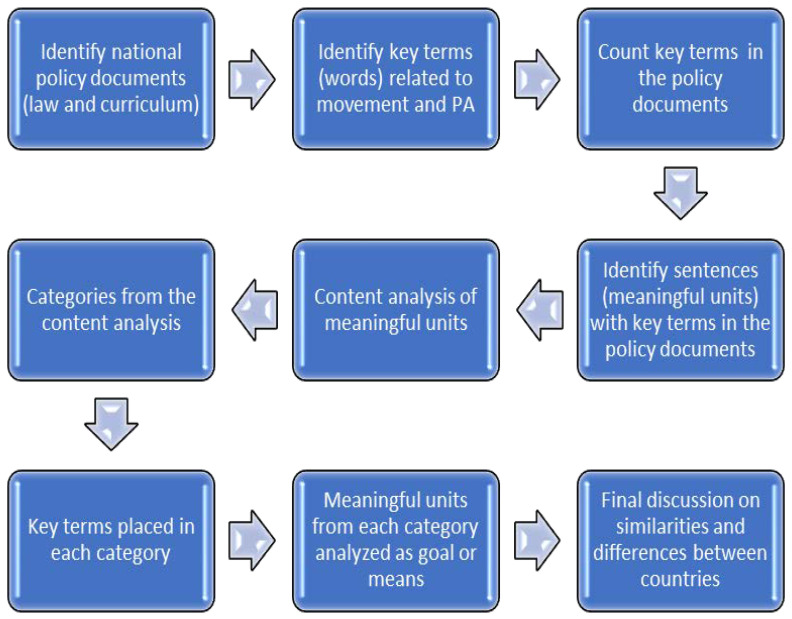
Flowchart depicting the steps in the research process.

A document analysis approach was employed to investigate the national policies of five Nordic countries to provide an overview of whether and how these documents position and value MoPA. Documents and artifacts are ready-made sources of data that are easily accessible and have the advantage of stability. The study was performed by a team of two researchers from each Nordic country specializing in MoPA among children and adolescents. Since the documents are written in each country’s language, and each country uses terms unique to its entity, the primary investigation was conducted by the researchers in their native language, drawing on their knowledge of any language-specific characteristics. In addition, the researchers shared a “coding frame” [50] during the data collection and analysis processes, which was an evolving document containing the identified keywords and the contexts in which they occurred. During the ongoing process of collecting data, the interplay between individual and group reflections was manifested through dialogue within the research group, both on the national level and among the whole group of 10 researchers from all five countries. The policy documents were analyzed in several steps (Figure 1).

First, the existing national policy documents (law and curriculum) in the five countries were obtained. Key terms (words) relevant to MoPA in these policy documents were next identified. A word-frequency analysis [40] was performed on each policy document (law and curriculum) to determine which key terms occurred in each national policy document and to what extent. As the policy documents were in five different languages, the key terms were identified in each language and then translated into English to facilitate clear, continuous discussions within the research group. The policy documents from each country were read carefully and thoroughly. Key MoPA-related terms contained in sentences were identified and defined as meaningful units. These meaningful units were collated and analyzed for similarities. Based on this analysis, categories were formulated. With this deeper understanding of the content, text passages assigned to each category were further compared to each other to ensure consistency within the categories. This research strategy represents a content analysis of documents to identify patterns in a replicable and systematic manner [51] and use these to generate categories according to meaning or connotation that facilitate further downstream analysis [52]. Both the word-frequency count and the content context review were performed independently by two researchers for each country. After comparing notes, the two researchers made a consolidated assessment to independently check the coding [53].

An analysis of the meaningful units from the categories was performed according to whether MoPA was expressed as a goal or a means. When MoPA was referred to for their own sake, it was considered to be being expressed as a goal. If MoPA was mentioned to reach other goals, it was categorized as a means. Finally, a consolidated assessment was conducted to assure that all steps were done in a similar manner in all countries. The resulting characteristics extracted from the five national curricula were compared and similarities and differences were identified and discussed.

## 3. Results

The results are presented under the three headings: “MoPA-related terms in ECEC law and curricula”, “Content related to MoPA in ECEC curriculum” and “MoPA as a goal or means in ECEC curriculum”. Under each heading, the similarities and differences between the Nordic countries are described.

### 3.1. MoPA-Related Terms in ECEC Law and Curricula

The word count analyses identified terms in the laws and curricula related to MoPA. The number of words in the documents differed between Nordic countries. The total word count for the law documents ranged from 1179 (Sweden) to 6414 (Finland) and, for the curricula, from 4860 (Denmark) to 13,933 (Iceland). In total, seven different MoPA-related terms were identified in the documents: bod* (e.g., body), coord* (e.g., coordination), íþrótt/liikunta* (a Nordic concept related to but not equal to sport), motor*, move* (e.g., movement), physic* activ* (e.g., PA) and physic* educ* (e.g., physical education) (Table 1).

In the law documents, the searched terms appeared infrequently in three countries’ documents (Denmark 2, Finland 2 and Iceland 2) and not at all in those from the other two (Norway 0 and Sweden 0). The four terms that occurred were bod* (Denmark 1 and Iceland 1), íþrótt/liikunta* (Finland 1), motor* (Iceland 1) and move* (Denmark 2 and Finland 1).

The MoPA-related terms appeared more frequently in the curricula than in the law documents. Three terms occurred in all five countries’ curricula (bod*, motor* and move*) and one term (physic* activ*) appeared in four countries (Finland, Iceland, Norway and Sweden). Two terms were mentioned by two countries: íþrótt/liikunta* and physic* educ* (Finland and Iceland), and one term, coordin*, was mentioned by two countries (Norway and Sweden). The terms that were most frequently present in the curricula across all five countries were move* (65 mentions), followed by bod* (41 mentions), íþrótt/liikunta* (22 mentions; 21 in Finland and 1 in Iceland), motor* (15 mentions), physic* activ* (13 mentions), physic* educ* (5 mentions) and coordin* (2 mentions).

### 3.2. Content Related to MoPA in ECEC Curriculum

The documents used for content analysis are the national curricula from the five countries. The laws were not included since the presence of MoPA-related terms were low. Content analyses based on the MoPA-related terms were used to identify the contexts surrounding these terms. These analyses resulted in the following context categories: Development, Environment, Expression, Health & Well-being, Learning and Play (Table 2).

Development. MoPA-related terms appeared in the context of development and were present in all five curricula, however in various ways. In all countries, children are encouraged to engage in different types of bodily movement experiences to affect many aspects of development especially, motor, social and personal development. All-round movements are intended to promote children’s active exploration of the world and the potential of their bodies, alone and together with other children, therefore also supporting children’s social interaction. Norway focuses on children’s development of motor skills, physical control, coordination, strength and agility. Denmark focuses on the ECEC staff as role models for finding joy in movement and to support and encourage children with limited experience with their body, senses and movement.

Environment. MoPA-related terms appeared in the context of the environment in all countries. In Denmark, Norway, Sweden and Finland, children should be supported by staff and the surrounding environment in experiencing the joy of movement. In Denmark and Norway, the outdoor and indoor spaces should be well suited for imagination and creativity. ECEC shall be an arena for daily PA, and it shall promote the joy of movement and motor development in children. Additionally, parents should be involved to secure good conditions for movement as well as provide movement challenges outside of ECEC. The Norwegian curriculum also highlighted that the staff should ensure that all children are active, and the Finnish curriculum also mentions cooperation from parents. In Iceland and Finland, the safety of the environment (indoor and outdoor) was also highlighted.

Expression. MoPA-related terms appeared in the context of expression in Denmark, Iceland, Finland, and Sweden. Expression in the Finnish curriculum has diverse meanings, such as different forms of art and culture, means of self-expression and communication, senses and the body as a research instrument. The Finnish curriculum does not include the aesthetic dimension, which is mentioned in Danish, Icelandic and Swedish curricula. In Denmark, sensory learning environments should handle aesthetic dimensions with a focus on children’s playful exploration and creative movement experiments, whereas the focus in the other countries is that children should be provided with a variety of different activities to be presented as different aesthetic means of expression.

Health & Well-being. MoPA-related terms appeared in the context of health and well-being in all countries and should improve when children are encouraged to use, challenge, experiment, feel and take care of their bodies through calmness and movement. In Finland, Sweden, Norway and Iceland, the focus is on the children gaining an understanding and/or knowledge of the importance of proper health (e.g., nutrition) and well-being including positive self-perception and exploring their own feelings/emotions but not in direct relation to MoPA. The Finnish curriculum also underlines the psycho-social safety issue.

Learning. MoPA-related terms appeared in the context of learning in four countries but are not mentioned in the Swedish curriculum. In Denmark and Norway, MoPA and/or using the body is central for learning. In Iceland, the joy of movement and/or physical education should be used to promote learning. In Finland, the focus on learning is a combination of academic skills (e.g., mathematical thinking, linguistic skills), as well as achieving knowledge on own body and general health aspects as well as the general promotion of the joy of movement.

Play. MoPA-related terms appeared in the context of play in the Finnish and Norwegian curricula and to some extent in the Swedish and Danish curricula. In Denmark and Norway, wild, adventurous, dangerous, and challenging games, and activities, referred to as “risky play”, were highlighted. In Finland, play is considered to be a child-oriented, creative, and natural way of learning.

### 3.3. MoPA as a Goal or Means in ECEC Curriculum

The meaningful units from the categories in the Nordic ECEC curricula were analyzed regarding whether MoPA was expressed as either goals or means. When MoPA was emphasized for its own sake, it was seen as a goal. If MoPA was emphasized for other areas, it was seen as a means. Table 3 uses excerpts to illustrate the distribution of goals and means throughout the five curricula. The MoPA-related terms were used both as goals and means in some variation in all five curricula. MoPA as a goal itself was described clearly and in detail in the Danish, Finnish, and Norwegian curricula and vaguely in the Icelandic and Swedish documents, which focused more on general means. In the detailed Finnish curriculum, MoPA was seen as an important learning element as well as a tool and method for learning academic skills. MoPA as a goal appeared in most of the identified context categories in the Danish and Norwegian curricula. In the Icelandic and Swedish curricula, MoPA was emphasized as a means for other areas of development to a large extent. MoPA development and learning were infrequently referred to as goals in the Icelandic and Swedish curricula, especially in the latter where MoPA was rarely formulated as a goal. The Icelandic curriculum contained short formulations that emphasized the importance of MoPA for health, social development, and interaction with the external world, while the Swedish curriculum focused on health, well-being and aesthetic development (Table 3).

## 4. Discussion

The present study focused on the value of MoPA in ECEC as embodied in the current laws and curricula of Nordic countries. Curriculum theory served as a framework, where the central object was education values, for example, what counts as knowledge, which knowledge is legitimized through curriculum formulations and the selection of knowledge for incorporation into a curriculum [27].

### 4.1. Similarities and Differences in Laws and Curricula

Findings showed that MoPA-related terms were present in Nordic curricula, but were not a part of the law, except on a few occasions in Denmark, Iceland, and Finland. Seven MoPA-related terms were identified in the policy documents: body, coordination, íþrótt/liikunta, motor, movement, PA, and physical education.

Some terms were used similarly by all Nordic countries, while others differed. Three terms (body, motor, and movement) occurred in all five countries and one term (PA) appeared in four countries (Finland, Iceland, Norway, and Sweden). The dominant term present in all curricula and the most used term in four out of five countries was movement. íþrótt/liikunta (a Nordic concept related to but not equal to sport) and physical education were only mentioned by Iceland and Finland, though the Nordic concept idræt/liikunta/íþrótt/idrett/idrott is common in general everyday language in all Nordic countries. The term “sport” was not mentioned in any of the laws nor in the curricula. The name of the school subject in Sweden and Denmark is “Idrott och hälsa” and “Idræt”, respectively, but was not mentioned in their ECEC policy documents. Coordination was only mentioned in the Norwegian and Swedish curricula. On the whole, minor parts of the curricula were devoted to MoPA, and MoPA-related terms represented a remarkably small part of the curricula relative to the total word counts of the governing documents, especially the laws. MoPA’s absence in law documents and its sparse occurrence in the curricula of Nordic countries indicates that MoPA is relatively low valued in these societies [27,28]. To increase the value of MoPA, which is important for health [10] and sustainability [6,7] from a lifelong perspective, [13,54,55], understanding and attitudes toward MoPA must be reconsidered.

The seven MoPA-related terms occurred in six different content contexts: development, environment, expression, health & well-being, learning and play. The same terms occurred in several contexts and variations within the same national curriculum and between different national curricula. MoPA-related terms appeared in the learning context in four of the five curricula but were completely absent from the fifth. The learning aspect of MoPA was not present in the Swedish curriculum. It is problematic that some knowledge areas are more valuable for children to learn than other areas. Inherent in most school curricula is some sort of curriculum hierarchy—that is, an assumption that some school subjects are more valuable than others [31,56]. Mathematics and language occupy a privileged position on top of the traditional curriculum hierarchy. They emphasize ‘abstraction from everyday life’, consist of a supposedly universal ‘language of ideas’ and are perceived to have clearly defined boundaries with established knowledge [57]. Ideas in society are reflected in curricula for school. However, the same phenomenon of curriculum hierarchy seems to be present in ECEC according to the findings of this study. That MoPA-related terms occurred in different content contexts may reflect the overall importance of MoPA, which seems to be a vague and low priority.

When MoPA is referred to for its own sake, it can be seen as a goal, while if the benefits of MoPA was emphasized in the context of other goals, it represents a means to another end [38]. MoPA was used as both a goal and a means in the documents analyzed. As a goal, MoPA was described clearly and appeared in detail in most of the identified context categories in the Danish, Finnish and Norwegian curricula, yet remained vaguely referenced in the Icelandic and Swedish curricula, where the focus was more general. In the Icelandic and Swedish curricula, MoPA was emphasized as a means of achieving other goals relatively frequently yet, references to development and learning MoPA as the goal itself were sparse in these curricula, especially in Sweden. If the matter is not clearly expressed as a goal it will hardly be interpreted and legitimized as important knowledge and practice [27]. The importance of knowledge in MoPA is often overlooked and interpreted as a natural part of development that the children undergo without the need for a specific focus on learning [58]. The conceptualization of MoPA as a natural part of development seems to dominate in all Nordic countries. However, there were differences between the five Nordic countries. Knowledge in MoPA was described as a goal and means in Denmark, Finland, and Norway, while it largely was described as a means for achieving other goals in Iceland and Sweden, which is an interesting observation that warrants further exploration in future studies.

Risk-oriented play was mentioned in the Danish and Norwegian curricula. Risky play assumes that the child is physically active in outdoor play and limitations on children’s play opportunities may be fundamentally hindering their healthy development [59]. The promotion of risky play may be a major agent in young children’s development and gaining physical and cognitive competencies as well as creativity, norms and self-efficacy [60,61]. Thorough safety efforts should be balanced with opportunities for children to develop physical competence. Safety issues were only mentioned in the Finnish curriculum. It is challenging for ECEC educators to promote opportunities in which children are allowed to choose freely and follow their interests in play even when these seem risky. Children engage in risky play predominantly when outdoors, but risks also present themselves indoors [62]. Risky play refers to physically active play as well as to FMS and knowledge in MoPA. The similarities and differences in the formulations of risky play and safety in the five Nordic curricula may reflect different societal values of children’s ability to develop physical competencies as well as norms and self-efficacy. There is an ongoing discussion about safety and whether today’s children are overprotected and never given the chance to develop the physical skills and self-confidence required to effectively cope with everyday risks in life. The different outcomes of those discussions can be seen in the five curricula. Overall, safety efforts must be comprehensive while still allowing opportunities for children to develop FMS and MoPA that support a lifelong PA perspective [63].

The foundations of FMS are laid in early childhood [8] and should, therefore, be emphasized in the curriculum for ECEC. The importance of movement is often overlooked because it is a natural part of human life [58]. Though it is crucial for the child’s general development [4,64,65], it is differentially emphasized and specified in the policy documents for ECEC in Nordic countries.

### 4.2. The Value of MoPA in ECEC

The sparse and diverse occurrence of MoPA-related terms gives the impression that MoPA is not as important as other matters in ECEC. Based on the low number and variety of MoPA-related terms used in policy documents relative to the total number of words, the diversity among the contexts in which the terms appear and the dissimilarity in the frequency with which MoPA is used as a goal rather than means, one is left to question whether MoPA is valued for ECEC in Nordic countries. According to Vallberg Roth (2014), learning the Nordic languages seems to be the most valued content in Nordic ECEC curricula [40]. Hännikäinen (2016) also concludes that language plays a crucial role in the learning/content areas specified in Nordic policy documents. Language and communication are perceived to be of utmost importance, potentially challenging the educators, while MoPA is infrequently mentioned and even hidden. It may thus appear that MoPA is given low priority in ECEC since what counts as legitimized knowledge is derived through policy formulations and the selection of topics in curriculum construction [33]. The sparse occurrence of terms related to MoPA does not motivate educators to implement MoPA in daily routines and thus influences practice in a negative way. Content selection processes are influenced by societal interests and trends. The final, agreed-upon curriculum is often what remains after a series of political, pedagogical, and ideological compromises [28,34,66] and is left up to the interpretation of educators in practice. Education is a field where struggles often occur between different actors, who are competing to define the field and ultimately to determine what knowledge is important [27,35]. Curricula should provide a guarantee that all children have the same and equal opportunity to develop an equivalent knowledge base, which includes MoPA, regardless of background and condition. Policy documents aim to guide teachers, politicians, and others to create a high-quality MoPA education for children in their most rapid physical development period. As the occurrence of MoPA-related terms is diverse and sparse, whether the curriculum guarantees that all children have an equal opportunity to develop motor competence and engage in PA remains debatable. Children who do not participate regularly in structured MoPA in the context of their homes may not have any access to MoPA in ECEC either if it is not valued in the curriculum. Children must learn FMS early in life, as these underlie sustainable physical fitness and a lifetime of rewarding, enriching PA [5]. Education in ECEC is vital because most children attend ECEC in the Nordic countries, which allows it to play a compensatory role. The WHO has reported that children of all ages around the globe are not physically active enough to fulfill the health recommendations [10]. This is a paradox as children have the right to develop to full potential and health [67].

Health outcomes from early childhood are essential for sustainability [6,7] and is included in the convention on the rights of the child [67]. While children must be physically active in the present so they can enjoy meaningful lives through play and engaging in joyful MoPA during their childhoods, MoPA is also important from a long-term perspective. Therefore, ECEC should be future-oriented and seen as a high-yield investment [68,69]. During the early childhood years, when most children attend ECEC in the Nordic countries, it is important to be physically active to a high extent, to develop their movement potential and to be physically literate [63]. Childhood development including FMS lays the foundation for a lifetime of mental and physical health as well as future education, productivity in working life and well-being [4].

Barriers to the implementation of FMS for public health benefits exist [70] and many children’s FMS are shown to be low [71,72]. Barriers arising from political and institutional curricular conflicts are drivers of the identified barriers preventing the adoption and implementation of FMS interventions for children [17]. Children’s developmental status, as well as the quality of care at home, parenting practices and access to ECEC, are used as indicators in UNICEF-supported surveys on children’s health around the world [6,7,73]. Theories of child development have served as the foundation for curriculum models., which reflect differences in values concerning what is important for young children to learn, as well as the process by which children are believed to learn and develop. These variations reveal the matters in focus and inform the role of teachers and how children should participate in learning [74]. Teaching competence in MoPA is crucial. Preschool-teacher education in the Nordic countries has been reformed in the past decade. There are variations among the countries, but all have placed an emphasis on strengthening education and moving it to a higher academic level [18]. Further investigations are warranted in future studies regarding MoPA in the Nordic countries, and within the countries’ higher education institutions.

The study also raises the question of who influences the formulation of policy documents. Since education is a field as other fields where struggles to define the field occur [27,35], those who ultimately formulate the documents are given preferential interpretation to what knowledge is deemed important and what should be included and given priority in the curriculum. Since this study shows differences in the policy documents studied, it could be of interest to examine who is given access to formulating these documents in the Nordic countries.

This study has some limitations. First, only binding policy documents were included in the investigation. In each country, additional manuals are available to guide educational practices. Second, law texts and curricula in their original native languages were used as these contain the most accurate representation of the content being analyzed. After the analysis, the results were translated into English. We considered the documents in their original language to be the most faithful and feared that information might be lost by translating these earlier in the analysis process. Third, the study involved ten researchers and continuous meetings were held to ensure and assess the research procedure. Despite these limitations, the present study contributes novel information regarding MoPA in the Nordic countries’ policy documents.

## 5. Conclusions

There is variation between Nordic policy documents for ECEC, but also some similarities. As the MoPA-related terms occurred infrequently relative to the total number of words contained in all five curricula and did not occur in the law documents in all countries, it can be concluded that the development of children’s MoPA is not prioritized in the policy documents for ECEC. For MoPA education to be equitable and effective, the matters comprising it must have sufficient intrinsic value in terms of knowledge and understanding to make them valued, pursued for its own sake and not solely as a means of achieving other goals. The sparse and diverse occurrence of MoPA-related terms, especially in the laws, but also in the curricula, actualizes the need to change the formulations to guarantee regular MoPA for all children in ECEC to ensure they develop to their full potential. Most children in the Nordic countries attend ECEC, which allows it to play a compensatory role in promoting MoPA. The WHO has reported that children of all ages around the globe are not physically active enough to fulfill the health recommendations, which is a paradox as children have the right to develop to full potential and health. Early childhood development is currently receiving increasing attention and is included in the Sustainable Development Goals by WHO and UNICEF. The findings from the present study support that MoPA, which is undeniably important for children’s development, health, and well-being, is a low priority value according to the Nordic policy documents delineating ECEC and the guidance provided therein to educators is inexplicit. Thus, the value of MoPA could be improved in the Nordic policy documents, which would benefit from being more specific and detailed so that ECEC staff with professional backgrounds can readily transform and integrate the content into the children’s daily practices. Future studies are warranted to investigate how different topics are valued in ECEC in the Nordic countries as well as to determine to what extent ECEC teacher education informs and prepares future ECEC teachers for using and teaching MoPA, but also, to what extent MoPA contributes to daily ECEC in Nordic countries.

## Figures and Tables

**Table 1:** The frequency of MoPA-related terms identified in policy documents from Denmark, Finland, Iceland, Norway and Sweden.

	Denmark		Finland		Iceland		Norway		Sweden		Total	
	Law	Curric	Law	Curric	Law	Curric	Law	Curric	Law	Curric	Law	Curric
Total word count	3588	4860	6414	13,275	3255	13,933	4457	8014	1179	5055	18,893	45137
Bod* (body)	1	16	0	11	1	5	0	8	0	1	2	41
Coordin* (coordination)	0	0	0	0	0	0	0	1	0	1	0	2
íþrótt/liikunta*	0	0	1	21	0	1	0	0	0	0	1	22
Motor*	0	4	0	3	1	2	0	2	0	3	1	14
Move* (movement)	2	18	1	19	0	17	0	6	0	5	3	65
Physic* activ* (PA)	0	0	0	6	0	2	0	2	0	3	0	13
Physic* educ* (physical education)	0	0	0	2	0	3	0	0	0	0	0	5
Total	3	38	2	62	2	30	0	19	0	13	7	162

*—Linguistic inflexions of the word.

**Table 2:** Terms appearing in the national curricula from Denmark, Finland, Iceland, Norway and Sweden and their context categories.

Term	Context Category	Country
Bod* (body)	Development	DK, FI, NO
	Environment	DK, FI, NO
	Expression	FI, IS
	Health & Well-being	DK, FI, NO, SE
	Learning	FI, NO
	Play	DK, FI, NO,
Coordin* (coordination)	Development	NO
	Health & Well-being	SE
íþrótt/liikunta*	Development	FI, IS
	Environment	FI
	Health & Well-being	FI
Motor* (motor…)	Development	DK, FI, NO
	Health & Well-being	FI, SE
	Learning	IS
	Play	SE
Move* (movement)	Development	DK, FI, IS, NO, SE
	Environment	DK, FI, IS, NO, SE
	Expression	DK, IS, SE
	Health & Well-being	DK, FI, NO
	Learning	DK, FI
	Play	FI
Physic* activ* (PA)	Development	FI, IS, NO, SE
	Environment	FI
	Health & Well-being	FI, IS, SE
	Learning	FI, IS
	Play	FI
Physic* educ* (physical education)	Development	FI
	Learning	FI, IS

*—Linguistic inflexions of the word.

**Table 3:** Excerpts reflective of MoPA as a goal and means in the national curricula from Denmark (DK), Finland (FI), Iceland (IS), Norway (NO) and Sweden (SE) according to context category.

Categories	Denmark	Finland	Iceland	Norway	Sweden
**Development**	Goal: “The pedagogical environment should support the stimulation of the three basic motor senses (…) which is crucial for the child’s motor development and automatization of movement (gross as well as fine functions)” Means: “The body is a source of awareness of other things and other people and one’s own body in the world, including in aesthetic, social, emotional and movement processes”	Goal: “Regular and supervised PA plays a key role in children’s holistic development and learning of motor skills” Means: “PA in a group develops children’s cognitive, social and emotional skills, such as interaction and self-regulations skills”.	Goal: No results Means: “PA positively affects children’s social interaction, their relationship to the external world, and their competence in both daily and novel situations”.	Goal:” Kindergarten shall be an arena for daily PA, and it shall promote the joy of movement and motor development in the children”. Means: “By engaging with this learning area (Body, movement, food and health), the children shall be enabled to use their bodies to sense, experience, play, learn and create”.	Goal: “Children should be given the opportunity to develop comprehensive mobility by being able to participate in physical activities and spend time in different natural environments”. Means: “Education should give children the opportunity to experience the joy of movement and thereby develop their interest in being physically active”.
**Environment**	Goal: No results Means: “Nature experiences during childhood have an emotional, a bodily, a social and a cognitive dimension”.	Goal: “PA refers to various kinds of activity with different levels of physical strain, including playing indoors and outdoors, field trips and supervised PA. In addition to supervised exercise, it is ensured that children get plenty of opportunities for independent PA both indoors and outdoors every day and season”. Means: “Different senses, as well as equipment made out of different materials encouraging children to be physically active, are utilized in the physical activities”.	Goal: “Preschool should provide a safe environment and space encouraging all children to engage in varied indoors and outdoors movement”. Means: “Playgrounds are an educational space with different landscapes, grounds and vegetation encouraging exploration, movement and expression”.	Goal: “…experience well-being, joy and achievement through a variety of physical activities, indoors and out, all year round”. Means: “Staff shall design the physical environment so that all children are given the opportunity to actively participate in play and other activities and so that toys and equipment are accessible to the children”.	Goal: No results Means: “promote a good, accessible environment for care, play, movement, development and learning,”
**Expression**	Goal: “Sensory learning environments should take account of the aesthetic dimension with focus on children’s playful exploration and creative movement experiments”. Means: No results	Goal: “The initiatives of younger children are often physical and non-verbal. Understanding and responding to these require sensitive presence and familiarity with the child”. Means: “Children are encouraged to consider and describe their mathematical observations by expressing and examining them, for example by using their body or different devices and images”.	Goal: “Preschools should encourage children’s interpretation and expression in varied ways and create space for play, dance and physical expression”. Means: No results	Goal: No results Means: No results	Goal: No results Means: “Education should give children the opportunity to experience, portray and communicate through different aesthetic forms of expression such as image, form, drama, movement, singing, music and dance”.
**Health & Well-being**	Goal: “The body is the source of mental health (e.g., well-being) as well as physical health (e.g., nutrition, hygiene, mobility”. Means: “Children exist in the world through their bodies, and the basis of physical and mental well-being is formed when they are encouraged to use, challenge, experience, feel and take care of their body through calmness and motion”.	Goal: “PA is children’s ways of being, basis of lifelong well-being together with guardians, children are also encouraged to exercise in their free time both indoors and outdoors”. Means: “Sufficient daily PA is important for the child’s healthy growth, development, learning and overall well-being”.	Goal: No results Means: “Daily PA as a basis for psychological, physical and social well-being and good health. Emphasis on challenging outdoor activities to enhance health and wellness. PA as part of a healthy lifestyle”.	Goal: “….feel confident in their own bodies, gain a positive view of themselves and explore their own feelings”. Means: “Kindergartens shall enable all of the children to discover the joy of movement, an appreciation for food and food culture, emotional and social well-being and good physical and mental health”.	Goal: No results Means: “When PA, nutritious meals and a healthy lifestyle are a natural part of children’s day, education can help children understand how this can affect health and well-being”.
**Learning**	Goal: “The pedagogical learning environment should support all children in experiencing the joy of movement and joy of their body, both in quiet and active situations so that the children feel comfortable with their bodies, including bodily sensations, body functions, senses and various forms of movement”. Means: “The pedagogical learning environment supports children’s general learning, including curiosity, drive, self-esteem and movement within and across the following themes: 1. Comprehensive personal development. 2. Social development. 3. Communication and language. 4. Body, senses and movement. 5. Nature, outdoor life and natural phenomena. 6. Culture, aesthetics and community”.	Goal: “In ECEC, children gather versatile experiences of different physical activities and games, such as traditional outdoor games as well as moving to stories and music”. Means: “Measuring is experimented with and the concepts of location and relation are practiced with the children, for example through games involving physical activities, by drawing or using different instruments”.	Goal: “Through physical education, the children learn about PA and develop motor skills”. Means: “Children enjoy a PA that promotes learning.”	Goal: “The children shall be able to use their entire body and all of their senses in their learning processes”. Means: use their bodies and senses to develop spatial awareness”.	Goal: No results Means: No results
**Play**	Goal: “There should be room for the “being” and “doing” of the body” Means: The outdoor space allows for bodily sensation, movement, imagination and creativity, and the ground is well suited for somersaulting and wild, adventurous and dangerous games and activities”.	Goal: “PA refers to various kinds of activity with different levels of physical strain, including playing indoors and outdoors, field trips and structured PA. Learning environments provide the children with alternatives for doing things that they enjoy, PA in versatile and fast ways, games and play ……”. Means: “They shall support children’s natural curiosity and desire to learn as well as guide them in play, be physically active, explore, express themselves through art as well as experience art”.	Goal: No results Means: “Play tests different competencies, and movement is an important part of it”.	Goal: “…evaluate and master risky play through physical challenges”. Means: “The children shall be included in activities in which they can engage in PA, play and social interaction and experience motivation and achievement according to their abilities”.	Goal: No results Means: “Play can also challenge and stimulate children’s motor skills, communication, collaboration, and problem-solving, as well as the ability to think in terms of images and symbols”.

## Appendix A

Nordic ECEC binding documents in native language, law and curricula, included in the study.

**Denmark**
*Law*
Dagtilbudsloven, kap 2 [Day-Care Facilities Act (2/2020): Chapter 2]
https://www.retsinformation.dk/eli/lta/2020/1326 (accessed on 14 October 2020)
*Curriculum*
Bekendtgørelse om pædagogiske mål og indhold i seks læreplaner [Executive Order on pedagogical objectives and content in six curriculum themes (968/2018)]
https://www.retsinformation.dk/eli/lta/2018/968 (accessed on 14 October 2020)
**Finland**
*Law*
Varhaiskasvatuslaki 540/2018
https://www.finlex.fi/fi/laki/alkup/2018/20180540 (accessed on 25 November 2020)
*Curriculum*
Varhaiskasvatussuunnitelman perusteet 2018
https://www.oph.fi/sites/default/files/documents/varhaiskasvatussuunnitelman_perusteet.pdf (accessed on 2 November 2020)
**Iceland**
*Law*
Lög um leikskóla 90/2008 [Preschool Act 90/2008].
https://www.althingi.is/lagas/nuna/2008090.html (accessed on 23 October 2020)
*Curriculum*
Aðalnámskrá leikskóla—Almennur hluti 2011 [The IcelandIc national curriculum—Guide for Preschools].
https://www.stjornarradid.is/media/menntamalaraduneyti-media/media/forsidumyndir/lokadrogleiksk_vefur.pdf (accessed on 23 October 2020)
**Norway**
*Law*
Lov om barnehager (Barnehageloven, 2021) [Act relating to kindergarten (The Kindergarten Act, 2021)].
https://lovdata.no/dokument/NL/lov/2005-06-17-64 (accessed on 16 November 2020)
*Curriculum*
Rammeplan for barnehagen (Kunnskapsdepartementet, 2017) [Framework Plan for Kindergartens (Ministry of Education and Research, 2017)].
https://www.udir.no/laring-og-trivsel/rammeplan-forbarnehagen/(accessed on 2 November 2020)
**Sweden**
*Law*
Skollag (2010:800). [The Education Act SFS 2010:800]
http://www.riksdagen.se/sv/Dokument-Lagar/Lagar/Svenskforfattningssamling/Skollag-2010800_sfs-2010-800/?bet=2010:800 (accessed on 1 September 2020)
*Curriculum*
Läroplan för förskolan: Lpfö -18. [Curriculum for the Preschool, Lpfö 18]
https://www.skolverket.se/undervisning/forskolan/laroplan-for-forskolan/laroplan-lpfo-18-for-forskolan (accessed on 1 September 2020)
